# Laboratory and Clinical Settings of Heavy/Light Chain (HLC) Assays in the Management of Monoclonal Gammopathies and Multiple Myeloma

**DOI:** 10.3390/jpm13050743

**Published:** 2023-04-27

**Authors:** Cecilia Napodano, Laura Ioannilli, Valerio Basile, Francesca Gulli, Valeria Carnazzo, Stefano Pignalosa, Luigi Di Biase, Erica Cavaleri, Cosimo Racco, Francesco Equitani, Mariapaola Marino, Umberto Basile

**Affiliations:** 1 Department of Laboratory Medicine and Pathology, S. Agostino Estense Hospital, 41126 Modena, Italy; cecilia.napodano@gmail.com; 2 Scientific Department, The Binding Site Italy, Part of Thermo Fisher Scientific, 24050 Bergamo, Italy; laura.ioannilli@bindingsite.com; 3 Clinical Pathology Unit and Cancer Biobank, Department of Research and Advanced Technologies, IRCCS Regina Elena National Cancer Institute, 00144 Rome, Italy; valeriobasile90@gmail.com; 4 Clinical Biochemistry Laboratory, IRCCS “Bambino Gesù” Children’s Hospital, 00165 Rome, Italy; dottfgulli@gmail.com; 5 Department of Clinical Pathology, Santa Maria Goretti Hospital, AUSL Latina, 04100 Latina, Italy; v.carnazzo@ausl.latina.it (V.C.); s.pignalosa@ausl.latina.it (S.P.); l.dibiase@ausl.latina.it (L.D.B.); e.cavaleri@auslo.latina.it (E.C.); c.racco@ausl.latina.it (C.R.); 6 Department of Transfusion Medicine and Immuno-Hematology, Santa Maria Goretti Hospital, AUSL Latina, 04100 Latina, Italy; f.equitani@ausl.latina.it; 7 Dipartimento di Medicina e Chirurgia Traslazionale, Sezione di Patologia Generale, Università Cattolica del Sacro Cuore, 00168 Rome, Italy; 8 Fondazione Policlinico Universitario “A. Gemelli” IRCCS, 00168 Rome, Italy

**Keywords:** multiple myeloma, free light chain, monoclonal component, heavy/light chain

## Abstract

The antibody-related immune response is mediated by immunoglobulins (Igs), soluble circulating glycoproteins produced by activated B cells that, upon the recognition of specific epitopes on pathogen surfaces, activate, proliferate, and differentiate into antibody-secreting plasma cells. Although the antibodies are effectors of the humoral immune adaptive response, their overproduction in response to a dysregulated proliferation of clonal plasma cell production in tumoral conditions (i.e., multiple myeloma), enriches the serum and urinary matrices, assuming the crucial role of biomarkers. Multiple myeloma (MM) is a plasma cell dyscrasia characterized by the expansion and accumulation of clonally activated plasma cells in bone marrow, determining the release of high amounts of monoclonal component (MC) that can be detected as intact immunoglobulin (Ig), immunoglobulin fragments, or free light chains (FLCs). The importance of detecting biomarkers for the diagnosis, monitoring, and prognosis of diseases is highlighted by the international guidelines that recommend specific assays for the analysis of intact Igs and FLC. Moreover, a developed assay called Hevylite^®^ allows for the quantification of immunoglobulins that are both involved (iHLC) and not involved (uHLC) in the tumor process; this is a fundamental aspect of following up the patient’s workup and evaluating the progression of disease, together with the treatments response. We here summarize the major points of the complex scenario involving monoclonal gammopathies and MM clinical management in view of advantages derived for the use of Hevylite^®^.

## 1. Introduction

A dysregulated proliferation of clonal plasma cells in bone marrow gives rise to a tumor burden as in multiple myeloma [1]. Multiple myeloma (MM) is a hematological malignancy characterized by the accumulation of clonally activated plasma cells in bone marrow, which are able to expand through their close interdependence with the surrounding microenvironment of the bone marrow [2] and to produce high amounts of monoclonal component (MC). MC can be detected as intact immunoglobulin (Ig), immunoglobulin fragments, or free light chain (FLC) either in the serum or urine [2,3,4]. The conventional assays used for the analysis of intact Igs are immunochemical dosages and serum protein electrophoresis (SPE) with quantification of the different electrophoretic zones, while immunofixation (IFE) is a qualitative method that allows for the typing of the MC [4]. Regarding the serum FLC, the International Myeloma Working Group (IMWG) included the FLC assay in combination with SPE and IFE for the diagnosis and monitoring of plasma cell dyscrasias [4]. The FLC assay can quantify an amount of FLC in serum, detecting extremely low levels of FLCk and FLCλ concentrations without showing significant cross-reactivity with bound light chains [4,5]. The use of the FLC assay significantly improved the diagnosis of monoclonal gammopathy, especially when the SPE results were inaccurate due to the CM migration zone; that is, when the electrophoretic migration overlapped with other proteins that migrate in the same area, such as β-lipoprotein or transferrin, generating outcomes that are hard to interpret [6]. In this context, new diagnostic approaches are being developed to facilitate the early management of lymphoproliferative diseases, permitting an improvement of the patient’s quality of life.

## 2. Overview of Monoclonal Gammopathies towards to Multiple Myeloma

**Figure 1:**
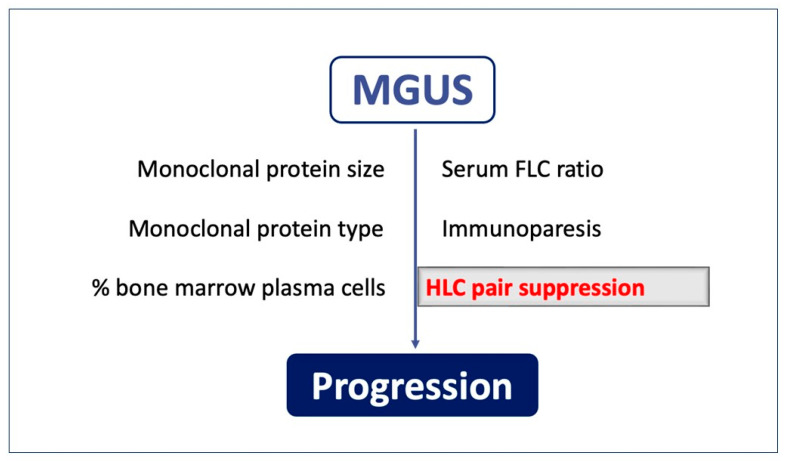
Immune biomarkers may be of relevance in predicting the risk of the progression of precursor conditions to multiple myeloma.

Abnormal B-cell clone expansion and the secretion of monoclonal proteins may indicate a clinically quiescent state for a prolonged period, defining monoclonal gammopathy of undetermined significance (MGUS), an asymptomatic monoclonal gammopathy [3,6] that may often display a worsening progression towards a malignant symptomatic lymphoproliferative disorder such as MM or Waldenström macroglobulinemia (WM) [4,7]. It is well known that MGUS carries a 1% average annual risk of progression, but it is also known that progression is highly variable amongst individuals. For this reason, it is important to identify risk factors for progression. In this context, different parameters must be considered, such as: the monoclonal protein’s size, type and quantity, bone marrow plasma cells, the serum FLC ratio, and immunoparesis. These biomarkers have a prognostic role, predicting the risk of progression from asymptomatic conditions, such as MGUS, to MM [8] (Figure 1).

Therefore, through these parameters, it is possible to conduct frequent monitoring of patients at higher risk, and those at lower risk can be reassured and followed less frequently [3,8]. Specifically, immunoparesis is defined by the suppression of polyclonal immunoglobulin levels and the degree of suppression of the uninvolved immunoglobulin heavy/light pair chain (uHLC) [8,9]. In this regard, another assay has been developed: the heavy/light-chain assay (HLC) (HevyLite^®^), which is able to detect the involved immunoglobulin heavy/light pair chain (iHLC) and the corresponding uninvolved immunoglobulin heavy/light pair chain (uHLC) [9]. Thus, this further tool allows for the discrimination of the non-tumoral/uninvolved Ig associated with the tumoral isotype: a phenomenon called isotype-matched immunoparesis (IMI) [9,10]. Therefore, the HLC assay has prognostic power in the MGUS risk stratification in the IgG and IgA MGUS sets [9], acquiring clinical importance in defining this pathologic area.

A wide range of clinical signs may be associated with the presence of monoclonal gammopathies [9,11,12]. Quiescent B-cell clones do not cause tumor symptoms, and even immunosuppression is uncommon. However, even a very small clone can induce severe manifestations due to the toxicity of the monoclonal immunoglobulin. It has been observed that MC may induce severe organ damage and dysfunction through its aggregation and deposition or through its antibody activity versus autoantigens, defining the group of monoclonal component-related diseases [13]. The main affected target organ is the kidney, defining the so-called monoclonal gammopathy of renal significance (MGRS) [12,13,14]. MGRS is associated with high morbidity due to the severity of renal and even systemic lesions induced by the monoclonal immunoglobulin component. Early diagnosis is crucial, as the suppression of monoclonal immunoglobulin secretion by chemotherapy often improves worsening outcomes. To better characterize the clinical settings caused by a “dangerous B-cell clone”, [13,15] a phenomenon that is still poorly recognized and frequently undertreated, researchers have proposed extending the concept of MGRS to that of monoclonal gammopathy of clinical significance (MGCS): this new term has been coined to include myriad conditions arising due to monoclonal pathogenic proteins [11,15]. The wide spectrum of MGCS depends on the involved organs and pathogenic mechanisms. Tissue damage, stemming from the deposition of monoclonal immunoglobulins, showed aggregate, amorphous, crystalline, microtubular, or fibrillar forms. Moreover, due to the deposition of immune complexes and activation of complement cascades, immune-mediated reactions against a tissue antigen are frequent mechanisms of damage in these disorders. This general scenario, including diverse expressions of monoclonal gammopathies, is centered on the presence of excessive monoclonal plasma cells in the bone marrow with damaged organs or tissues in the case of full-blown multiple myeloma. Until a few years ago, the diagnosis of MM was undertaken on a clinical basis alone, with the recognition of CRAB signs: an acronym that defines a state of hypercalcemia, renal insufficiency, anemia, and bone lesions [10,11,16].

Recently, the international guidelines for the definition of MM have been updated, identifying valid biomarkers, in addition to the CRAB criteria, in patients who would otherwise be considered as having smoldering multiple myeloma (SMM): an intermediate condition between MGUS and MM without CRAB signs, as wells as MGUS, but with higher levels of clonal bone marrow plasma cells (≥30 g/L) [4,16]. MM patients display symptoms of end-organ damage in the presence of one or more biomarkers, a monoclonal component > 30 g/L, and at least 10% clonal bone marrow plasma cells/biopsy or extramedullary plasmacytoma, which are known as the SLiM criteria [4]. Among these biomarkers, serum free light chains (FLC) have been included outside the reference range (sFLC involved/uninvolved ratio ≥ 100-rFLC) with the involved FLC ≥ 100 mg/L [4]. The rFLC together with clonal bone marrow plasma cells ≥ 60% and two or more MRI focal lesions represent the myeloma defining events (MDE).

As suggested by Mateos et al., the treatment of asymptomatic high-risk patients leads to a decrease in the rate of malignant transformation and improvement in overall survival [17]). Early diagnosis results in fewer complications in the presentation of symptomatic disease [18], although most plasma cell clones with different features and genetic abnormalities have been identified in the same patient suffering from MM. By comparing the genetic characteristics of the cells involved in the different phases of the natural history of the disease (from diagnosis to relapse), in about half of cases, the clone highlighted at relapse is genetically different from the clone present at diagnosis [19]. This development has significant implications, as asymptomatic patients with active disease can now be considered for treatment before the onset of potential irreversible organ damage.

## 3. Development of Multiple Myeloma

In the development of MM from MGUS and/or smoldering MM (SMM), a wide range of events take place involving both the plasma cell clone and the bone marrow microenvironment. Among these, heterogeneous genetic lesions, interactions between myeloma cells in the bone marrow niche, osteoblasts, osteoclasts, and components of the immune system, mediated by the hyperproduction of cytokines and growth factors, play a crucial role, together with alterations in bone remodeling and the bone marrow angiogenesis process [19,20].

This biological and genetic heterogeneity relates to the difficulty of identifying laboratory and clinical parameters with defined prognostic roles. In fact, most of the factors still used in clinical practice no longer have a predictive role after the introduction of new drugs. The reason for the poor prognostic reliability of both the clinical and biological parameters is the persistence of residual clonal myeloma cells in patients who have experienced complete remission after chemotherapy and/or stem cell transplantation [21].

The introduction of novel therapeutic strategies including autologous stem cell transplantation changed the natural history of the disease, leading to new manifestations of relapse. Although MM is characterized by the proliferation of an abnormal plasma cell clone, the genetic heterogeneity of the disease is both interclonal and intraclonal [22]. This biological heterogeneity represents the major feature of clonal evolution and of the disease’s progression and eventual relapse [19], and it often results in considerable clinical differences. In fact, some patients show an aggressive pathology and an unfavorable prognosis, while others show indolent clinical behaviors with longer life expectancies.

Based on this evidence, mandatory molecular events are not achieved in a linear way for the development of MM, but through branching or non-linear pathways, typical of the evolutionary model proposed by Darwin [19,23]. This model of MM development is based on the idea that mutations are acquired randomly and are selected according to the clonal advantages they confer. An excellent tool for studying the global impact of intraclonal heterogeneity is provided by monitoring the type of immunoglobulin produced and secreted at relapse (either a whole immunoglobulin or a light chain or both), enabling clinicians to make the adequate therapeutic decisions without delay. One of the most important goals of clinical research is to correlate the heterogeneous clinical outcomes with different biological features. The assumption in this model is that patients display different clones with different secretory behaviors: one clone can produce a complete Ig, whereas the other clones may secrete only an FLC, and this can be used as a marker for subclonal progression [23]. Increased immunoglobulin FLCs and an abnormal FLC ratio are observed in MM, and they have prognostic significance. An abnormal FLC ratio (involved/uninvolved FLC ≥ 100) is one of the MDEs included in the updated diagnostic criteria for newly diagnosed MM [4] and is an accurate predictor of survival and therapeutic responses in MM [24,25]. In fact, an abnormal FLC ratio before autologous stem cell transplantation (ASCT) predicted early progression, while a one-third reduction in 30–60-day post ASCT was a predictor of superior outcomes [24,25]. In the last decade, the Food and Drug Administration (FDA) has approved a new immunoassay that is able to measure the intact immunoglobulin (heavy/light chains—HLC), which improves sensitivity for monitoring disease response and prognosis [24,25,26], helping to collect more insights to define the features of the malignancy.

## 4. Heavy/Light Chains

**Figure 2:**
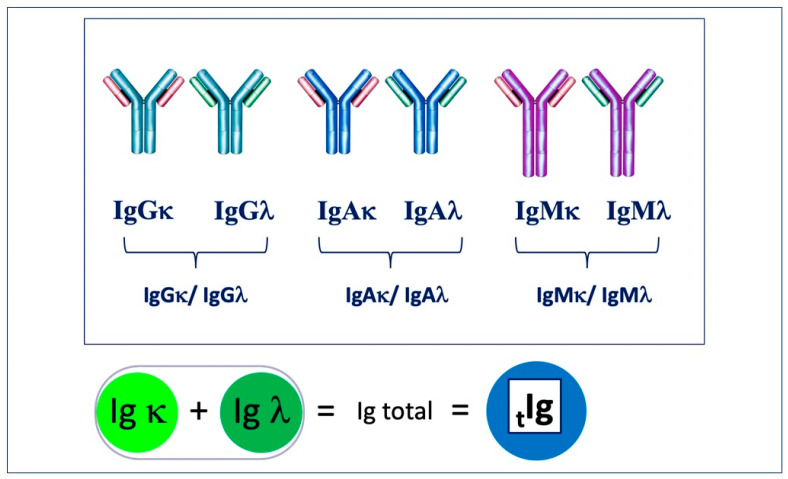
This assay is capable of separately identifying and quantifying the k and λ light-chain Igs associated with the same isotype, and this sum should be similar to the total immunoglobulin.

In 2009, a new immunoassay to measure heavy/light chains (HLCs) was introduced [26,27]. Immunoglobulin heavy/light-chain (HLC) assays separately quantify the different light-chain types of each immunoglobulin subtype (IgGκ, IgGλ, IgAκ, IgAλ, IgMκ, and IgMλ) and related Ig′κ/Ig′λ ratios. This assay can separately identify and quantify the k and λ light-chain Igs associated with the same isotype for the first time (Figure 2).

**Figure 3:**
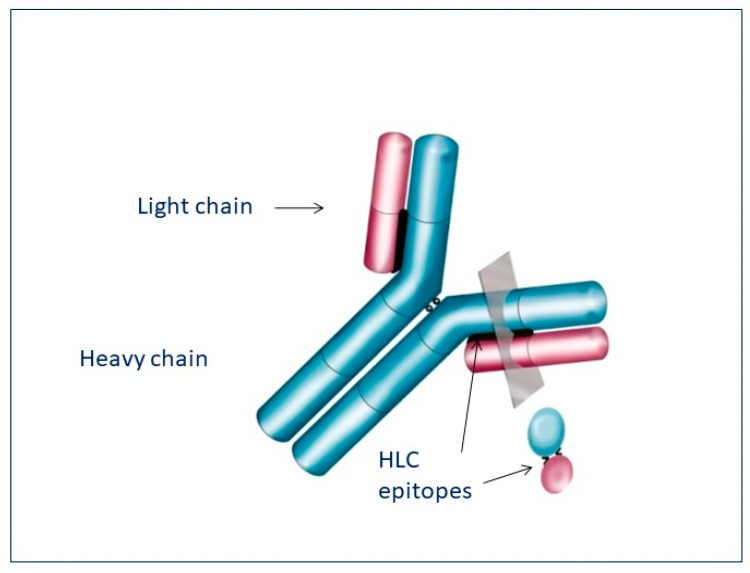
The polyclonal antibodies in the Hevylite test are targeted against unique epitopes found at the constant region’s junction of the heavy chain and the light chain.

Furthermore, this assay exploits the ability of antibodies to bind directly to target epitopes at the junctional region between bound k or λ light chains and their respective heavy-chain molecules [28] (Figure 3).

Ig chains are assessed in pairs to produce the HLC ratio in the same manner as the κ/λ sFLC ratio, being an indicator of clonal expansion [5,10]. HLC assays provide an alternative tool to aid in the management of diseases associated with monoclonal plasma cell proliferative disorders, encompassing a broad spectrum of diseases ranging from monoclonal gammopathy of undetermined significance (MGUS) [10,11] to more complex cases of multiple myeloma (MM) [10,11,29]. In this scenario, Hevylite highlights more specific insights into the evaluation of each tumor clone if compared with the total Ig quantification performed using conventional techniques [5,28] (Figure 2).

The sensitivity of the HLC ratio (rHLC) in detecting monoclonal intact immunoglobulins is dependent upon the concentration of both the involved (monoclonal) and uninvolved (polyclonal) immunoglobulin HLC of the same isotype, which also provides accurate information about isotype-matched immunoparesis (IMI) [5,10]. The different parameters evidenced by the HLC assay are summarized in Table 1.

Abnormalities in HLC are associated with various clonal or reactive B-cell disorders, including plasma cell disorders, B-cell lymphoma, and B-cell hyperplasia after immune stimulation; preliminary multivariate analysis indicates that the IgMκ/IgMλ HLC ratio is predictive of PFS as it is exhibited in multiple myeloma [30]. The assessment of the uninvolved HLC pair shows a possible polyclonal immunosuppression (IMI—isotype matched immunoparesis), which is predictive of plasma cell disorders; this suppression might be present in a significant number of patients with lymphoma. Therefore, HLC allows for the evaluation of the immunosuppression index through the quantification of the levels of uninvolved isotype counterparts [10]. The heavy/light chain assay represents a valid supportive method in the clinical management of MM and related disorders, since it allows for an accurate measurement of the antibody isotype involved in the malignant processes (iHLC) and uninvolved isotypes (uHLC) [31].

The Guidelines of the International Myeloma Working Group (IMWG), together with scientific reports, state that the HLC test has an important role to play throughout the management of a patient with MM, from diagnosis to prognosis and up to the assessment of the response to therapy [31,32,33,34].

## 5. Advantages and Limits of the Hevylite Assay

The Hevylite assay provides additional key data for diagnosing patients with MM. Compared to serum protein electrophoresis (SPE), this assay is more sensitive, less laborious, processes a quantitative result avoiding the subjective interpretation of the gel, and can overcome many of the known limitations of the SPE, such as co-migration and migration diffusion of the monoclonal component (MC), dye saturation, and non-linearity [32,34,35,36]. The major disadvantage of SPE is its inability to distinguish between monoclonal and polyclonal Igs, which is particularly important during follow-up assessments because the worsening of the disease is associated with an increase in MC and a decrease in polyclonal Igs. Nevertheless, regarding diagnostic practice, SPE remains part of the response criteria [32].

## 6. The Role of Hevylite in the Depth of Response in IgA-MM Patients

The IMWG guidelines for the standard investigative work-up of patients state that, “For patients with measurable monoclonal protein in serum, both electrophoretic studies and quantitative immunoglobulins are recommended to assess response, although electrophoretic measurements to follow monoclonal protein are preferred” [37]. However, in many cases, IgA monoclonal proteins are non-quantifiable by serum protein electrophoresis. In several studies, the percentage of non-quantifiable samples from IgA patients was typically 44% [35,38,39]. For many of these samples, the co-migration of the monoclonal IgA protein with other serum proteins in β-region (i.e., transferrin) [39] prevents accurate quantitation of the M-spike by SPE; for this reason, the IMWG recognize the limitations of electrophoresis, and state that “For several patients, especially with IgA or IgD myeloma, nephelometric quantitation of serum immunoglobulin is necessary” [37]. However, clonal IgA chain quantification by nephelometry or turbidimetry, which naturally comprises monoclonal and polyclonal immunoglobulins, does not accurately reflect the tumor burden. For this reason, there is a need to find novel indicators that more accurately reflect the disease burden and the response to treatment, and correlate to patients’ outcomes in myeloma with the IgA isotype (about 30–40% of patients) [39]. In the retrospective study conducted by Boyle et al., oligosecretory MM patients were identified by an M-component < 10 g/L thanks to rHLC, which was abnormal in all patients. Interestingly, among the IgA myeloma series, 51% of patients were evidenced through SPEP, but an extra 47% of patients became measurable when showing an abnormal rHLC. Therefore, it has been possible to accurately quantify up to 98% of igA-MM patients through Hevylite [39].

## 7. The Response Assessment: rHLC and dHLC

The quantitative measurement of total Igs is also recommended by the IMWG criteria [32], but, although accurate, this technique is unable to distinguish between MC and the polyclonal background. Interestingly, the deep detection of iHLC and uHLC enables us to discriminate the evolution of monoclonal gammopathies and the response to treatment through two methods of comparison: the HLC ratio (rHLC) and HLC difference (dHLC). In multiple myeloma, preliminary reports indicate that rHLC is a factor for predicting progression-free survival (PFS) [32,40], but several findings highlight the pivotal role of HLC, together with the FLC immunoassay, in diffuse large B-cell lymphoma (DLBCL) [30]. The measurement of iHLC shows a linear correlation with MC quantification, and the iHLC/uHLC ratio represents a marker of tumor clonality [30,41]. Several studies reported that 97% of patients with IgA- or IgG-type MM have an altered iHLC/ uHLC ratio at diagnosis [41,42] and that there is an association between the presence and diagnosis of suppression of uninvolved immunoglobulin (uHLC) and shorter survival [33,42], as well as a higher incidence of infection [33,42,43,44].

The IMWG criteria for the therapy response and definition of minimal residual disease (MRD) recognize the Hevylite method as an additional assay for the management and monitoring of patients with oligosecretory MM (monoclonal component < 10 g/L) or with an IgA MC migrating in the β-zone of electrophoretic trace [32]. Therefore, the IMWG acknowledges the potential clinical value of HLC measurements as a prognostic tool and states that: “the use of the heavy/light-chain ratios might have an important role in the definition of a minimal residual disease-negative state” [32].

Several studies, in fact, have also shown that rHLC could indicate residual disease in MM patients with normalized SPE after therapy, allowing for relapse detection earlier than traditional methods [32,43,45,46,47,48]. Ludwig et al. demonstrate that rHLC was the first proof of relapse in MM patients relapsing only intact immunoglobulins without any abnormal serum FLC ratios (FLCr) [35]. This evidence encourages the monitoring of both FLC and HLC criteria, analyzing the evolution of clones secreting diverse types of MCs [35,49].

Through periodic Hevylite monitoring, Ludwig et al. detected the presence of disease in patients who achieved a complete response (CR) with traditional methods and the conversion of the iHLC ratio. Normal to abnormal uHLC has been shown to be an early biomarker of disease progression [35], based on the quantification of the uninvolved uHLC isotype as a putative biomarker of the response to therapy. In this regard, three groups have been identified: (1) the absence of suppression; (2) moderate suppression (uHLC values below normal limits and an iHLC/uHLC ratio decrease up to 50%); (3) severe suppression (uHLC values below the limits and an iHLC/uHLC ratio decrease above 50%) [35]. The persistence of severe suppression during the follow-up of patients with MM is associated with a shorter survival period, while the value normalization in patients who have achieved at least one very good partial response (VGPR) shows a good response to treatment and immune system recovery [33,35]. The normalization of uHLC with a polyclonal production recovery is related to the response degree. Michallet et al. evaluated the impact of new therapies in the reconstitution of the polyclonal structure, which was almost always below the normal level: up to 97% of patients had uHLC suppression at diagnosis. Autologous stem cell transplantation (ASCT), together with maintenance therapy with Lenalidomide, positively influenced the recovery of the polyclonal structure, improving outcomes and survival compared to controls (no transplant) [43]. The Hevylite test in combination with other serological markers (sFLC, IFE) could provide useful information on the efficacy of the treatment, and the normalization of HLC values represents a valid surrogate marker for the evaluation of MRD, before proceeding to a bone marrow harvest [10,43,50]. Fouquet et al. focus on the HLC response to therapy, comparing standard IMWG response criteria [32] with the HLC criteria and evaluating the response assessment of rHLC changes [51].

**Figure 4:**
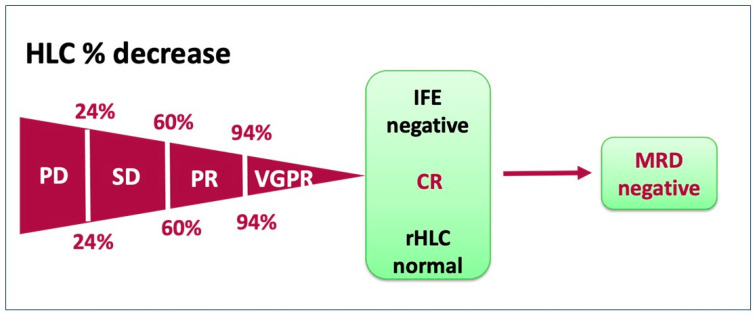
Representation of different response grades to therapy.

As shown in Figure 4, a very good partial response (VGPR) is defined by a >94% reduction in the rHCL from the baseline; a partial response (PR) is defined by a reduction in the rHCL from the baseline between 60% and 94%; stable disease (SD) is defined by a change in the rHCL from baseline < 24% increase but <60% reduction; and progressive disease (PD) is defined by a 24% increase in the rHCL from the baseline. A complete response (CR) is obtained with a negative immunofixation (IFE) or an rHLC within the normal range (1.12 to 3.21 for IgGj/IgGk; 0.78 to 1.94 for IgAj/IgAk) [51] (Figure 4). The analogue HLC criteria are defined by dHLC changes with a similar scale: PD is indicated by an increase of >25%, SD by a minor decrease to 50%, PR by a reduction between 50 and 90%, and VGPR by a reduction ≥ 90%. Nevertheless, the CR is obtained by a bone marrow infiltration < 5% and a normal rHLC with absent plasmacytomas [10,51], highlighting the pivotal role of ratio and difference as comparison methods.

## 8. Hevylite Pair Suppression

As a tumor grows, there is often a reduction in polyclonal plasma cells and, consequently, polyclonal immunoglobulins. Thus, the measurement of a Hevylite pair that is the same immunoglobulin class but the alternate light-chain type to that produced by the tumor (uHLC) results in a reduced concentration. When the concentration of the uHLC is below the normal range and an abnormal rHLC is present, it is referred to as HLC pair suppression [44].

It is possible to obtain important information about tumor clonality from the relationship between iHLC and uHLC. In addition, the detection of uHLC values lower than the normal range with an altered iHLC/uHLC ratio provides important information regarding the level of suppression of the immune system, which is induced by the myeloma itself [31].

The mechanisms of suppression of normal Igs in plasma cell dyscrasias remain poorly understood: uHLC suppression below normal levels and an altered iHCL/uHLC ratio have been observed to be associated with an increased risk of progression to myeloma in patients with MGUS [9,28,31,34,52]. Katzmann et al. developed a risk stratification model based on the serum concentration of MC (>15 g/L), the level of uHLC suppression, the sFLC ratio, and the isotype involved in the tumor process (IgA or IgM). The authors identified five groups based on the risk factors present (0, 1, 2, 3, 4), and the likelihood of progression to MM increases with the measurement of these biomarkers [52]. A recent paper confirms the particular importance of HLC measurement in the management of patients with MM [28].

The greater prevalence of IMI (immunoparesis matched isotype) over the suppression of uninvolved isotypes (classical immunoparesis), along with the lower proportional values for IMI, supports an isotype specificity of early suppression mechanisms for IgG and IgA isotype smoldering multiple myeloma (SMM). Immunoparesis is associated with other recognized risk factors for progression, but, especially in case of IgM, it appears to develop in advanced disease and could correspond to different suppression mechanisms. These mechanisms are involved at the time of the initial evaluation and during the follow-up assessments of patients with SMM using the serum Hevylite assay [52,53].

Altered values of HLC indicate the presence of an early relapse compared to traditional tests, associating with a lower survival rate of MM patients and a likely increased progression from MGUS to MM. Together with the free light-chain test and other serum markers, the Hevylite test guarantees the excellent monitoring of patients with plasma cell dyscrasias [54]. Interestingly, a multivariate analysis conducted by Jimenez and colleagues demonstrates that severe IMI, rather than classical immunoparesis, is an independent risk factor of progression from MGUS to MM [54,55]. Thus, the IMI is an additional risk factor that is able to discriminate between high-risk MGUS patients [52,55].

Recently, the effect of HLC pair suppression has been studied as a risk factor for bloodstream infections (BSI) and early death in a Spanish clinical trial, which included 115 newly diagnosed intact immunoglobulin MM patients with measurable disease [44]. The BSI are recurrent and are associated with a high mortality rate; thus, infections are the most serious threats to these patients [56]. This study is consistent with previous studies, which demonstrated that MM patients reported a mortality rate of 10% within 60 days of diagnosis [56,57]. Specifically, MM patients are susceptible to infections because their immune systems are weakened either by their advanced age, the disease, or the effects of chemotherapy. Consequently, having the opportunity to predict the risk of infection and early mortality for these causes would enable us to examine the immune system’s attempt to control the tumor [44]. This novel study showed that patients with severe HLC-matched pair suppression had a higher risk of BSI than patients without suppression, defining the IMI a promising factor for determining the risk of infection in MM patients. In contrast, classical immunoparesis, which is called systemic immunoparesis and defined by the suppression of one or two of the non-involved immunoglobulins, was not significantly associated with the risk of infection. Finally, the results suggest that identifying patients with IMI could help hematologists to choose tailored therapeutic strategies to minimize the risk of infection and early death. Therefore, the clinical role of HLC-matched pair suppression as a biomarker of bacterial bloodstream infection and the early mortality in MM patients has been clarified, even if the same role in viral infections remains to be investigated in further projects [44].

## 9. Conclusive Remarks

In the general context of monoclonal gammopathies, the FLC assay represents the progenitor among the biomarkers in the analysis of MC variation. Nevertheless, a recently developed assay called Hevylite^®^ is capable of quantifying both the immunoglobulins involved in the tumor process and the immunoglobulins that are not involved (polyclonal) with relative comparisons analysis (rHLC and dHLC) [35]. The correct quantification of MC during the disease’s progression is fundamental to evaluating the treatment’s response [35,58] and, thus, making the most appropriate treatment decisions. The Hevylite assay, cited by the IMWG guidelines, provides rapid and accurate results, which play an important role during baseline diagnosis, predicting the patient’s prognosis and outcome, and monitoring their response to treatment [29].

Interestingly, the Hevylite assay has also been widely applied in other diagnostic areas, since the identification of sensitive and specific biomarkers is crucial for prognostic diseases analysis. For instance, in patients with chronic hepatitis C virus, related mixed cryoglobulinemia (HCV-related MC), and increased risk of B-cell non-Hodgkin Lymphoma (B-NHL), HLC may represents a serum biomarker that is able to identify patients with overt B-NHL associated with mixed cryoglobulinemia vasculitis in chronic HCV infection [54,59]. The correlation between post-treatment HLC values shows a discrepancy between clinical and laboratory remission and the serological markers assessments, which remained substantially above the normal range and were unaffected by RTX therapy, displaying clonality persistence and the presence of possible MRD. Their employment could be useful for recognizing patients who could benefit from additional anti-CD20 therapy, with an improvement in patient-tailored treatments [60].

Therefore, biomarkers play pivotal roles in the evaluation and measurement of specific parameters in physiological processes, pathogenesis, and response to therapy. In this context, Hevylite, with other serum assay biomarkers, might display a predictive and prognostic ability to improve treatment outcomes, by which patients could receive multiple competing drug options or other treatment administrations modalities (i.e., precision surgery, radiotherapy). Thus, the diagnostic potential of innovative assays helps to implement the paradigm of precision medicine: ‘the right treatment to the right patient at the right time’, adding, “the right assay to the right patient at the right time”.

## Figures and Tables

**Table 1:** HLC nomenclature.

Term	Definition	For an IgAk Patient
HLC ratio	e.g., IgAk/IgAλ	IgAk/IgAλ
iHLC	Involved heavy + light chain isotype	IgAk
uHLC	Uninvolved heavy + light chain isotype	IgAλ
dHLC	iHLC–uHLC	IgAk–IgAλ
HLC pair Suppression	Whent the concentration of the uHLC is below the normal reference interval	Reduced concentration of Igaλ

## Data Availability

The original data presented in the study are included in the manuscript. Further requests should be addressed to the corresponding author.

## Abbreviations

Igs: immunoglobulins; MC: monoclonal component; SPE: serum protein electrophoresis; IFE: immunofixation electrophoresis; FLC: free light chain; HLC: heavy/light chain; MM: multiple myeloma; MGUS: monoclonal gammopathy of undetermined significance; SMM: smoldering multiple myeloma; WM: Waldenström macroglobulinemia; MGRS: monoclonal gammopathy of renal significance; IMI: immunoparesis matched isotype; iHLC: involved immunoglobulin heavy/light pair chain; uHLC: uninvolved immunoglobulin heavy/light pair chain; HLC: heavy/light-chain ratio; dHLC: heavy/light-chain difference; MDE: myeloma-defining events; CRAB: calcium, renal impairment, anemia, bones lesion; PFS: progression-free survival; ASCT: autologous stem cell transplantation; FDA: Food and Drug Administration; HCV-related MC: chronic hepatitis C virus—related mixed cryoglobulinemia; DLBCL: diffuse large B-cell lymphoma; IMWG: International Myeloma Working Group; MRD: minimal residual disease; PD: progressive disease; SD: stable disease; PR: partial response; VGPR: very good partial response; CR: complete response. SliM: plasma cells (S), light chains (li), magnetic resonance imaging (M).
